# Local Surgicel/rhBMP-7 delivery is associated with renal repair responses after surgically induced acute kidney injury

**DOI:** 10.3389/fmed.2026.1941191

**Published:** 2026-09-09

**Authors:** Ante Jakšić, Dragica Bobinac, Vedran Micek, Tanja Ćelić Črnac, Mirna Bobinac, Marin Oštrić, Josip Španjol, Kristina Pilipović

**Affiliations:** 1 Department of Anatomy, Faculty of Medicine, University of Rijeka, Rijeka, Croatia; 2 Department of Urology, Clinical Hospital Center Rijeka, Rijeka, Croatia; 3 Institute for Medical Research and Occupational Health, Zagreb, Croatia; 4 Department of Anesthesiology, Reanimatology, Emergency and Intensive Care Medicine, Faculty of Medicine, University of Rijeka, Rijeka, Croatia; 5 Clinic for Anesthesia, Intensive Care Medicine and Pain Management, Clinical Hospital Center Rijeka, Rijeka, Croatia; 6 Department of Surgery, Faculty of Medicine, University of Rijeka, Rijeka, Croatia; 7 Department of Cardiothoracic Surgery, Clinical Hospital Center Rijeka, Rijeka, Croatia; 8 Department of Urology, Faculty of Medicine, University of Rijeka, Rijeka, Croatia; 9 Department of Basic and Clinical Pharmacology and Toxicology, Faculty of Medicine, University of Rijeka, Rijeka, Croatia

**Keywords:** bone morphogenetic protein 7, ischemia-reperfusion injury, kidney injury, renal fibrosis, renal repair

## Abstract

Surgically induced acute kidney injury (AKI) may involve both ischemia–reperfusion and direct mechanical damage to the renal parenchyma, potentially affecting subsequent tissue repair and remodeling. Although bone morphogenetic protein-7 (BMP-7) has shown renoprotective effects in experimental models of acute kidney injury, most studies have evaluated systemic administration in relatively homogeneous injury models. The effects of local BMP-7 delivery in the setting of combined ischemic and surgical parenchymal injury remain insufficiently defined. In this study, a rat model combining transient renal ischemia–reperfusion with a standardized parenchymal incision was used to investigate renal repair following surgical injury. Recombinant human BMP-7 (rhBMP-7) was applied locally to the injured renal parenchyma using Surgicel as a carrier. Serum renal function measures, tubular injury, proliferative activity, fibrosis-associated remodeling, and selected repair- and BMP-associated signaling markers were evaluated at 7 and 28 days after injury. Local Surgicel/rhBMP-7 application was associated with lower serum blood urea nitrogen and creatinine levels at day 7 compared with the suturing-only surgical-injury group. Histopathological tubular injury was observed in both groups, without significant between-group differences. Increased numbers of proliferating cell nuclear antigen (PCNA)-positive tubular epithelial cells were observed in the Surgicel/rhBMP-7 group at both analyzed time points. Compared with the suturing-only group, the Surgicel/rhBMP-7 group showed higher Wnt-4 immunoreactivity at both time points and higher phosphorylated Smad1/5/8 immunoreactivity at day 28, compatible with differences in BMP-associated signaling. Histological analysis demonstrated a reduced collagen-positive area in the Surgicel/rhBMP-7 group, accompanied by a time-dependent pattern of *α*-SMA expression. In conclusion, local Surgicel/rhBMP-7 delivery was associated with differences in functional, tubular proliferative, repair-associated signaling, and histological remodeling responses following combined renal ischemia–reperfusion and surgical parenchymal injury. These findings support further evaluation of this localized delivery approach in more comprehensively controlled models of surgical kidney injury.

## 1 Introduction

Acute kidney injury is characterized by a rapid decline in renal function accompanied by structural damage (1). Although AKI is potentially reversible, incomplete or maladaptive repair may lead to persistent tubular injury, interstitial fibrosis, and progression to chronic kidney disease. The balance between regenerative and profibrotic signaling pathways is therefore a critical determinant of outcome after renal injury (2).

Bone morphogenetic protein-7 plays an important role in renal epithelial homeostasis and post-injury repair (3, 4). Experimental studies have demonstrated that exogenous BMP-7 can attenuate fibrosis and improve functional recovery in models of ischemic, obstructive, and toxic kidney injury (5–7). However, much of the available evidence derives from relatively homogeneous experimental injury models and systemic BMP-7 administration.

Surgically induced AKI represents a distinct experimental and pathophysiological setting in which ischemia–reperfusion may occur together with direct mechanical injury to the renal parenchyma. During nephron-sparing procedures, temporary hilar clamping may expose renal tissue to transient ischemia followed by reperfusion, while parenchymal resection and subsequent reconstruction produce additional direct surgical injury. Hemostatic materials may also be applied locally to the resection or reconstruction site (8). The resulting combination of ischemic and mechanical injury differs from conventional experimental models based on isolated ischemia–reperfusion and may influence subsequent tissue repair and remodeling. The present model was designed to reproduce key components of this combined surgical injury by incorporating transient renal ischemia together with standardized parenchymal incision and suturing. Despite its clinical relevance, localized BMP-7 delivery in this context remains insufficiently characterized.

In addition, most available data on BMP-7 focus on systemic delivery, whereas the biological responses associated with local application directly to an exposed parenchymal injury site remain less well defined (9, 10). Local delivery may therefore be particularly relevant in renal surgical settings, where the injured parenchyma is directly accessible during the operative procedure.

Therefore, the aim of this study was to investigate renal repair responses associated with local Surgicel/rhBMP-7 delivery in a rat model combining transient renal ischemia–reperfusion with standardized surgical parenchymal injury. We hypothesized that local Surgicel/rhBMP-7 delivery to the parenchymal injury site would be associated with differences in early and late renal repair responses compared with suturing alone.

## 2 Materials and methods

### 2.1 Animals

Male Wistar rats (3 months old; 250–300 g) were obtained from the Institute for Medical Research and Occupational Health (Zagreb, Croatia). All procedures were conducted in accordance with national legislation (NN 135/06, 37/13, 125/13, 55/13, 39/17) and Directive 2010/63/EU, and were approved by the institutional ethics committee and the Croatian Ministry of Agriculture. The study was designed as an exploratory experimental model, and the number of animals was minimized in accordance with the principles of Replacement, Reduction, and Refinement (3Rs).

### 2.2 Experimental model

**Figure 1:**
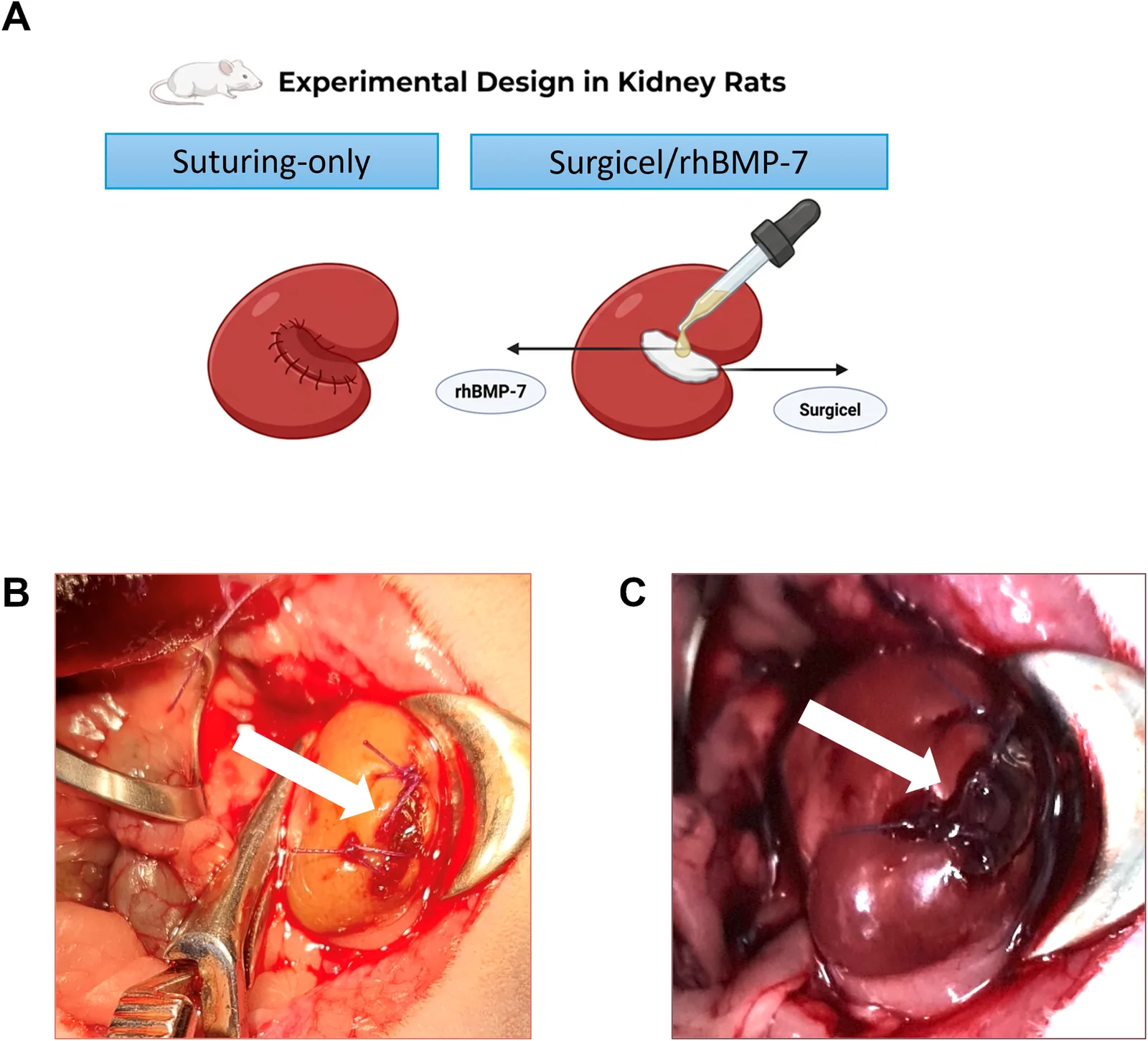
Experimental design and surgical procedure. **(A)** Schematic representation of the experimental model combining transient ischemia and parenchymal incision. **(B)** *In vivo* image of the kidney before declamping of the renal artery. **(C)** *In vivo* image of the kidney after declamping. The injury site is indicated by an arrow.

Animals were anesthetized with ketamine (80 mg/kg) and xylazine (12 mg/kg) administered intraperitoneally. Following a midline laparotomy, the left kidney was exposed and the renal artery was clamped for 10–15 min to induce transient ischemia. A standardized wedge-shaped incision (5 mm in length and 3 mm in depth) was created along the lateral border to induce direct parenchymal injury (Figure 1). Thus, this experimental model combines transient renal ischemia with direct parenchymal trauma, representing a surgically induced AKI model that captures both ischemic and mechanical injury components.

A total of 28 animals were used in the study and were equally distributed between the two experimental groups and the two time points. Animals were assigned to two groups: (1) suturing alone and (2) suturing with local Surgicel/rhBMP-7 application. In the Surgicel/rhBMP-7 group, rhBMP-7 (stock concentration, 0.1 mg/mL; MyBioSource, San Diego, CA, USA) was diluted to a concentration of 2 µg/mL. A piece of Surgicel (Ethicon Inc., Raritan, NJ, USA), trimmed to approximately 5 × 3 mm to match the dimensions of the parenchymal defect, was soaked in 0.2 mL of the prepared rhBMP-7 solution. The amount of rhBMP-7 used for Surgicel preparation corresponded to 0.4 µg per kidney. The prepared Surgicel was then placed directly into the parenchymal injury site, followed by suturing of the defect. A single administration was selected to reflect an intraoperative local treatment applied directly to the exposed parenchymal injury site. Following renal artery declamping, the kidney was returned to the abdominal cavity and the abdominal incision was closed in layers.

The local administration strategy and dose were selected on an exploratory basis, informed by previous studies demonstrating biological activity of exogenous BMP-7 in experimental renal injury models and by experimental evidence supporting local application of BMP proteins in tissue-remodeling settings (10–12).

Serum BUN and creatinine were measured in three animals from each group at each time point. For tissue-based analyses, kidneys from these three animals were used for histological and immunohistochemical analyses, whereas kidneys from the remaining four animals per group at each time point were used for Western blot analysis.

### 2.3 Tissue collection

At days 7 and 28 after surgery, animals were anesthetized with ketamine (80 mg/kg) and xylazine (12 mg/kg) administered intraperitoneally and euthanized by exsanguination via transection of the cervical blood vessels. Animals allocated to histological and immunohistochemical analyses were transcardially perfused with 0.9% saline followed by 4% paraformaldehyde (PFA) in 0.1 M phosphate buffer (pH 7.4). Kidneys were harvested, further fixed in 4% PFA for 24 h, and embedded in paraffin. Animals allocated to Western blot analysis were not perfused with PFA; their kidneys were harvested, frozen in liquid nitrogen, and stored at −80 °C until analysis.

### 2.4 Histology, histochemistry and imaging

#### 2.4.1 Hematoxylin and eosin (H&E) staining and histopathological evaluation

Paraffin sections (3 µm) were stained with hematoxylin and eosin according to standard protocols. Histopathological evaluation was performed by an observer blinded to group allocation using light microscopy (Olympus BX51, Olympus, Japan). Tubular injury was assessed based on tubular dilatation, epithelial cell necrosis, and loss of brush border. Injury was semi-quantitatively graded in randomly selected, non-overlapping cortical fields as follows: 0 (none), 1 (<25%), 2 (25%–50%), 3 (50%–75%), and 4 (>75%).

#### 2.4.2 Mallory's trichrome staining

Fibrotic changes were evaluated using Mallory trichrome staining. Collagen-positive area was quantified by threshold-based color segmentation and expressed as a percentage of total tissue area.

#### 2.4.3 Immunohistochemistry staining and image analyses

Paraffin-embedded sections (3 µm) were deparaffinized, rehydrated, and subjected to heat-induced antigen retrieval (citrate buffer, pH 6.0). Endogenous peroxidase activity was blocked with 3% hydrogen peroxide, followed by incubation in blocking solution. Sections were incubated overnight at 4 °C with primary antibodies (Supplementary Table S1) against *α*-SMA, PCNA, Pax2, Wnt-4, BMP-7, and phosphorylated Smad1/5/8, followed by appropriate HRP-conjugated secondary antibodies. Immunoreactivity was visualized using DAB and counterstained with hematoxylin.

Images were acquired using an Olympus BX51 microscope equipped with a DP70 digital camera and analyzed using ImageJ software (NIH, Bethesda, MD, USA). PCNA- and Pax2-positive nuclei were manually counted in randomly selected cortical fields at × 400 magnification and expressed as the number of positive cells per field.

For Wnt-4, BMP-7, and phosphorylated Smad1/5/8, DAB signal intensity was quantified in proximal tubules. Regions of interest were manually defined, and optical density was calculated after color deconvolution and background subtraction using the formula OD = log (255/mean intensity). Results were expressed as mean OD per pixel².

α-SMA expression was quantified as the percentage of positively stained area using threshold-based segmentation.

### 2.5 Creatinine and urea nitrogen assay

For renal function analysis, blood samples were collected via retro-orbital puncture on days 7 and 28 after surgery, and the supernatant was obtained after centrifugation at 3,000 rpm for 10 min to obtain rat serum. Blood serum was collected to assess the concentration of blood urea nitrogen (BUN) and serum creatinine (sCr), using commercially available kits (Creatinine Assay Kit and Urea Assay Kit; Abcam, Cambridge, UK).

### 2.6 Western blotting

Renal cortical tissue was homogenized in lysis buffer supplemented with protease and phosphatase inhibitors. Homogenates were centrifuged at 10,000 × g for 10 min at 4 °C, and the supernatants were collected for protein analysis. Equal amounts of protein were separated by SDS–PAGE and transferred to nitrocellulose membranes. Membranes were blocked in 5% non-fat dry milk and incubated overnight at 4 °C with primary antibodies (Supplementary Table S1), followed by appropriate secondary antibodies conjugated with horseradish peroxidase.

Signals were detected using a chemiluminescent substrate (SuperSignal West Pico, Thermo Fisher Scientific, Waltham, MA, USA) and visualized using a Kodak Image Station 440CF. Images were acquired and stored as original BIP (raw) files containing the full dynamic range of the detected signal. Representative blot images shown in the figures were contrast-adjusted for visualization purposes only. Target-band densitometry was performed on the original nonsaturated images using Kodak 1D Image Analysis Software. Total-protein signal in each corresponding Ponceau S-stained lane was quantified using ImageJ, and target-band intensity was normalized to the total Ponceau S signal of the respective lane. Protein loading and transfer efficiency were verified by Ponceau S staining, and densitometric values were normalized accordingly. Full-length uncropped blots and corresponding Ponceau S staining are provided in the Supplementary Information.

For collagen I analysis, the lower-molecular-weight immunoreactive band (approximately 70 kDa) detected with the COL1A (COL-1) monoclonal antibody (sc-59772, Santa Cruz Biotechnology, Supplementary Table S1) was used for densitometric quantification.

### 2.7 Statistical analyses

Data were collected using the Microsoft Excel (Microsoft Corp., Redmond, WA, USA) and, when necessary, corrected for between-session variation as described previously (13). Statistical analyses were performed using the GraphPad Prism 10 (GraphPad Inc., San Diego, CA, USA). The statistical test used for each dataset is specified in the corresponding figure legend. Results are expressed as means ± standard deviations (SD). In all comparisons, *p* < 0.05 was considered to indicate statistical significance.

## 3 Results

### 3.1 Renal function and tubular injury after surgical kidney injury

**Figure 2:**
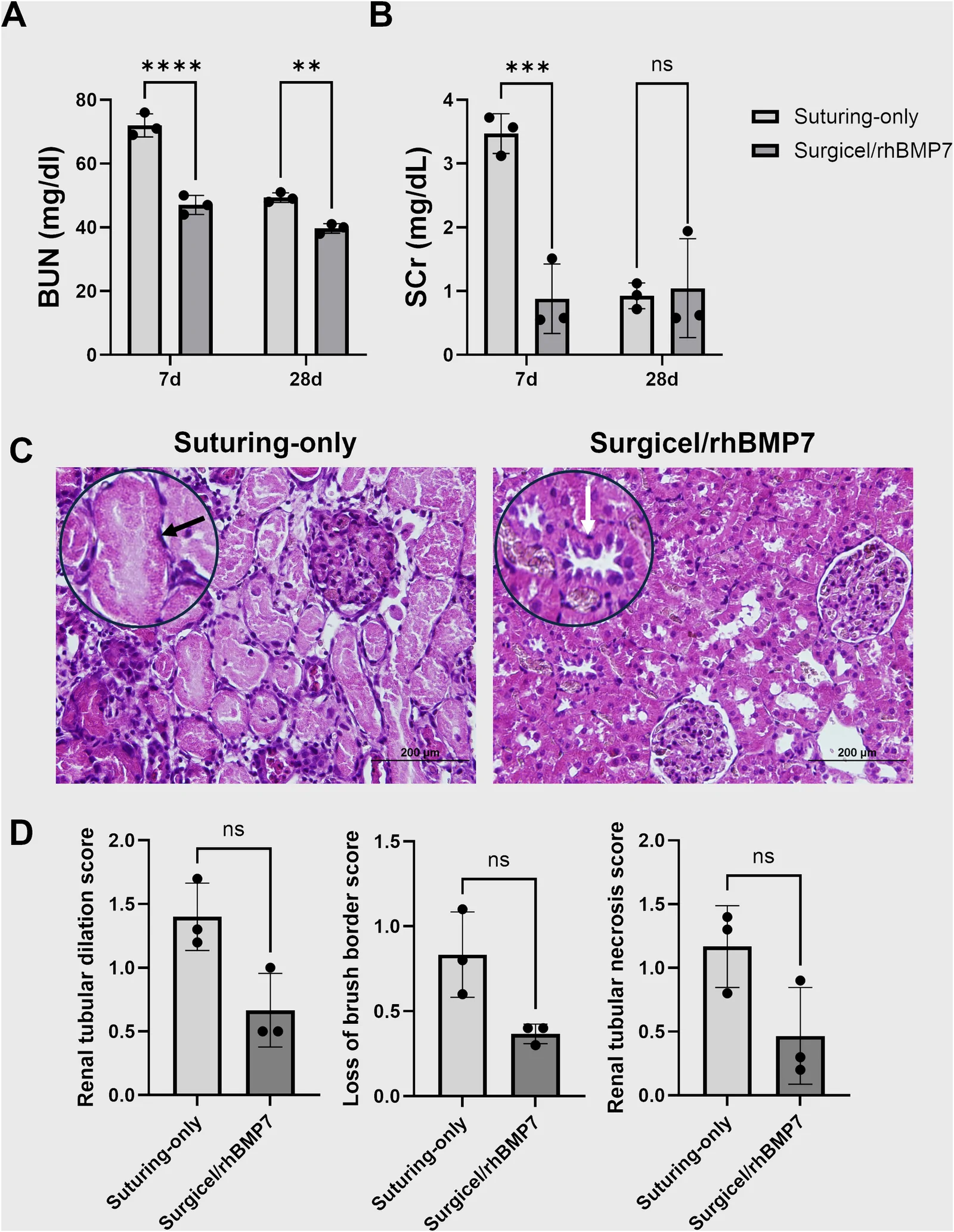
Renal function and histopathological tubular injury after combined renal ischemia–reperfusion and surgical parenchymal injury. Serum levels of **(A)** blood urea nitrogen (BUN) and **(B)** serum creatinine (sCr) were measured at 7 and 28 days after injury. BUN and sCr concentrations are expressed in mg/dL. Data are presented as mean ± SD (*n* = 3 animals per group at each time point). Statistical significance for renal function was assessed using two-way ANOVA with Šídák’s multiple-comparisons test: **p < 0.01, ***p < 0.001, ****p < 0.0001; ns, not significant. **(C)** Representative hematoxylin and eosin (H&E)-stained kidney sections from the suturing-only and Surgicel/rhBMP-7 groups at 7 days after injury. In the suturing-only group, the black arrow indicates tubular injury characterized by loss of brush border, tubular dilatation, and intraluminal cellular debris. In the Surgicel/rhBMP-7 group, the white arrow indicates a tubule with preserved epithelial morphology and less prominent luminal debris in the representative field. Scale bar, 200 µm. **(D)** Semiquantitative histopathological assessment of tubular dilatation, loss of brush border, and tubular necrosis at 7 days after injury. Data are presented as mean ± SD (*n* = 3 animals per group). Comparisons between the suturing-only and Surgicel/rhBMP-7 groups were performed using the Mann–Whitney U test; ns, not significant. BUN, blood urea nitrogen; sCr, serum creatinine; rhBMP-7, recombinant human Bone Morphogenetic Protein 7.

At day 7, serum BUN and sCr levels were lower in the Surgicel/rhBMP-7 group than in the suturing-only group (BUN, *p* < 0.0001; sCr, *p* = 0.0005) (Figures 2A,B). By day 28, both parameters were lower than at day 7 in both groups. No significant between-group difference in sCr was observed at day 28, whereas BUN remained lower in the Surgicel/rhBMP-7 group. Representative H&E-stained sections illustrated tubular dilatation, loss of brush border, and tubular necrosis in both groups. Although these alterations appeared less prominent in representative sections from the Surgicel/rhBMP-7 group, semiquantitative scores did not differ significantly between groups (Figures 2C,D).

### 3.2 Renal tubular epithelial proliferation and repair markers

**Figure 3:**
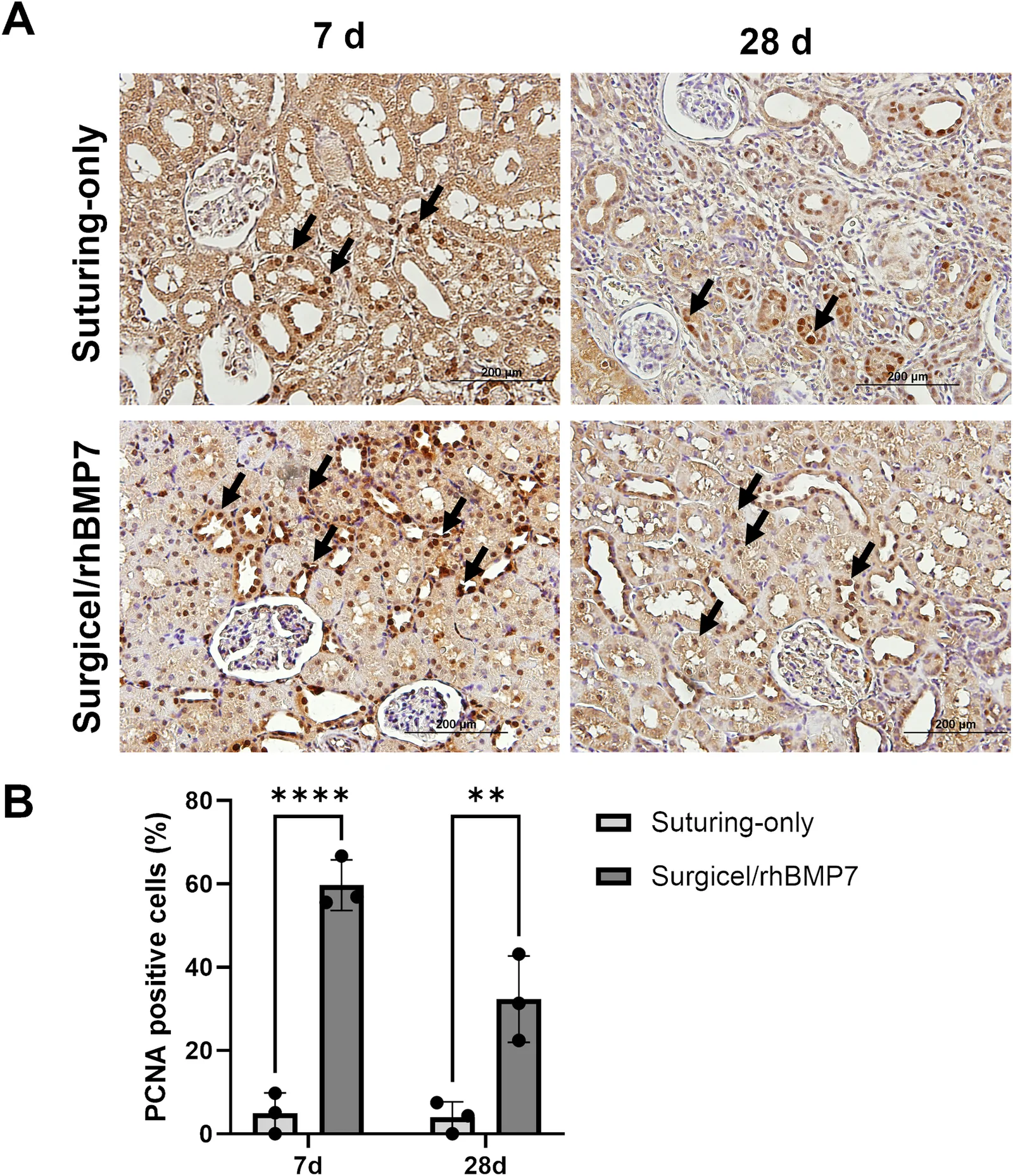
Tubular epithelial proliferation assessed by PCNA immunohistochemistry. **(A)** Representative images of rat kidney sections stained for proliferating cell nuclear antigen (PCNA) in the suturing-only group and the Surgicel/rhBMP-7-treated group at 7 and 28 days after injury. PCNA-positive nuclei are indicated by arrows. Scale bar: 200 μm. **(B)** Quantitative analysis of PCNA-positive cells in kidney tissue at 7 and 28 days after injury. Data are presented as mean ± SD (*n* = 3 animals per treatment group at each time point). Statistical significance: **p < 0.01, ****p < 0.0001 compared with the suturing-only group at the corresponding time point (two-way ANOVA with Šídák’s multiple comparisons test). PCNA, proliferating cell nuclear antigen; rhBMP-7, recombinant human Bone Morphogenetic Protein 7.

PCNA immunostaining revealed low numbers of PCNA-positive tubular epithelial cells in the suturing-only group at both time points (Figures 3A,B). The Surgicel/rhBMP-7 group showed higher numbers of PCNA-positive cells at day 7 (*p* < 0.0001), which remained higher than in the suturing-only group at day 28 (*p* = 0.0018), although the number was lower than at day 7.

**Figure 4:**
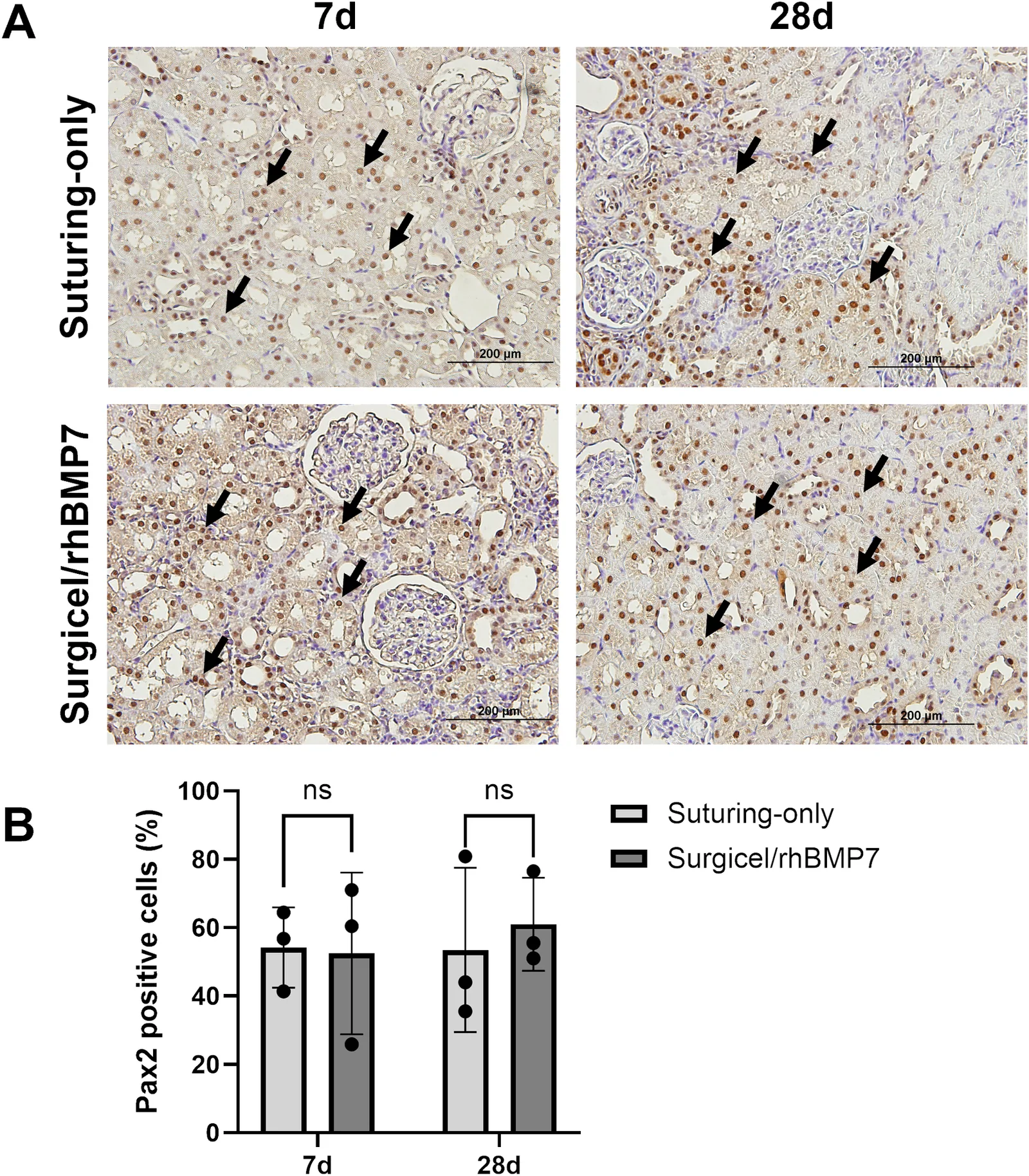
Pax2 expression following surgically induced acute kidney injury. **(A)** Representative images of rat kidney sections stained for Pax2 in the suturing-only group and the Surgicel/rhBMP-7-treated group at 7 and 28 days after injury. Pax2-positive nuclei are indicated by arrows. Scale bar: 200 μm. **(B)** Quantitative analysis of Pax2-positive cells in kidney tissue at 7 and 28 days after injury. Data are presented as mean ± SD (*n* = 3 animals per treatment group at each time point). Statistical analysis was performed using two-way ANOVA with Šídák’s multiple comparisons test. ns, not significant. Pax2, Paired box gene 2; rhBMP-7, recombinant human Bone Morphogenetic Protein 7.

Pax2-positive cells were detected in tubular compartments of both the suturing-only and Surgicel/rhBMP-7 groups at days 7 and 28 (Figure 4A). Quantitative analysis revealed no significant between-group differences in the proportion of Pax2-positive cells at either time point (day 7: *p* = 0.9925; day 28: *p* = 0.8718) (Figure 4B).

**Figure 5:**
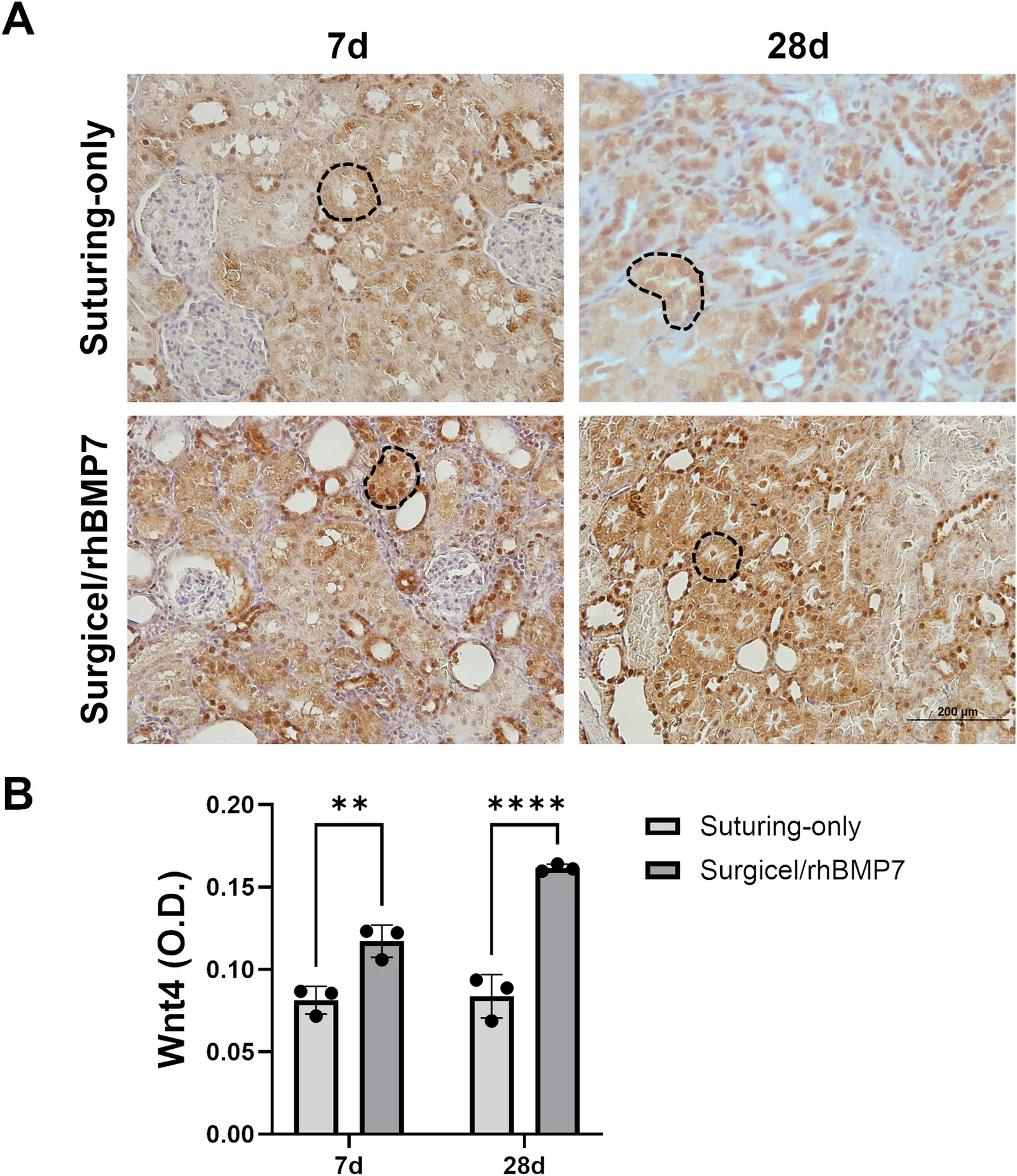
Wnt-4 expression in renal tubular compartments after injury. Representative brightfield images of Wnt-4 immunohistochemistry **(A)** and quantification of staining **(B)** in the suturing-only group and the Surgicel/rhBMP-7-treated group at 7 and 28 days after injury. Proximal tubules are outlined (dashed lines). Quantification was performed after DAB color deconvolution and conversion to optical density (OD) within proximal tubular ROIs following background subtraction. Data are presented as mean ± SD (*n* = 3 animals per treatment group at each time point). Statistical significance: **p < 0.01, ****p < 0.0001 compared with the suturing-only group at the corresponding time point (two-way ANOVA with Šídák’s multiple comparisons test). Scale bar: 200 µm.

Wnt-4 immunoreactivity was detected in the suturing-only group at both time points, without a significant temporal difference (Figures 5A,B). Compared with the suturing-only group, Wnt-4 immunoreactivity was higher in the Surgicel/rhBMP-7 group at day 7 (*p* = 0.0030) and day 28 (*p* < 0.0001).

**Figure 6:**
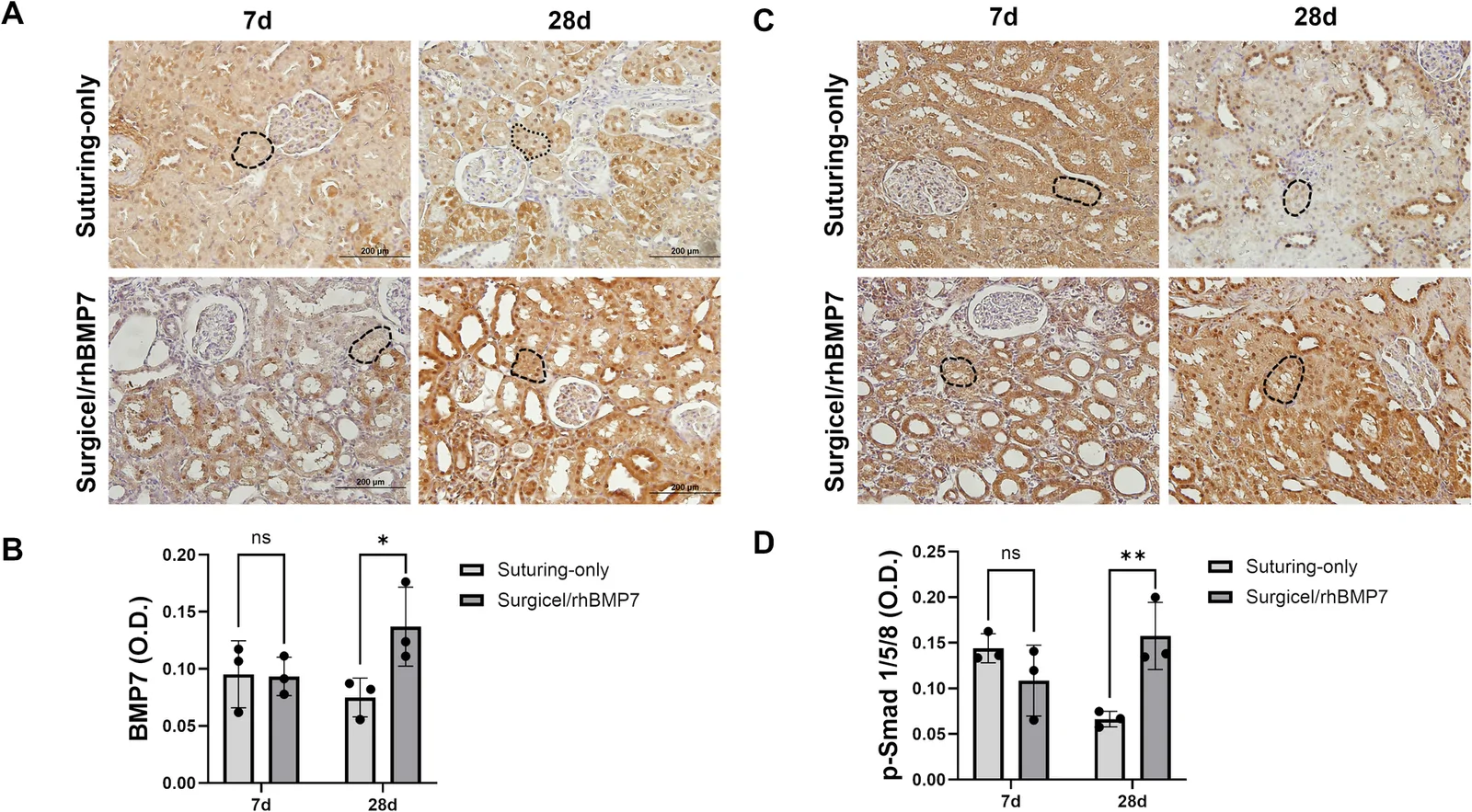
BMP-7 and phosphorylated Smad1/5/8 immunoreactivity after surgically induced acute kidney injury. Representative brightfield images of BMP-7 **(A)** and p-Smad1/5/8 **(C)** immunohistochemistry and quantification of staining **(B,D)** in the suturing-only group and the Surgicel/rhBMP-7-treated group at 7 and 28 days after injury. Proximal tubules are outlined (dashed lines). Quantification was performed after DAB color deconvolution and conversion to optical density (OD) within proximal tubular ROIs following background subtraction. Data are presented as mean ± SD (*n* = 3 animals per treatment group at each time point). Scale bar: 200 µm. Statistical significance: *p < 0.05, **p < 0.01, ns, not significant, compared with the suturing-only group at the corresponding time point (two-way ANOVA with Šídák’s multiple comparisons test). rhBMP-7, recombinant human Bone Morphogenetic Protein 7.

BMP-7 immunoreactivity did not differ significantly between groups at day 7 (*p* = 0.9954), whereas higher BMP-7 immunoreactivity was observed in the Surgicel/rhBMP-7 group at day 28 (*p* = 0.0359) (Figures 6A,B). Phosphorylated Smad1/5/8 immunoreactivity was also higher in the Surgicel/rhBMP-7 group than in the suturing-only group at day 28 (*p* = 0.0085) (Figures 6C,D).

### 3.3 Fibrotic remodeling

**Figure 7:**
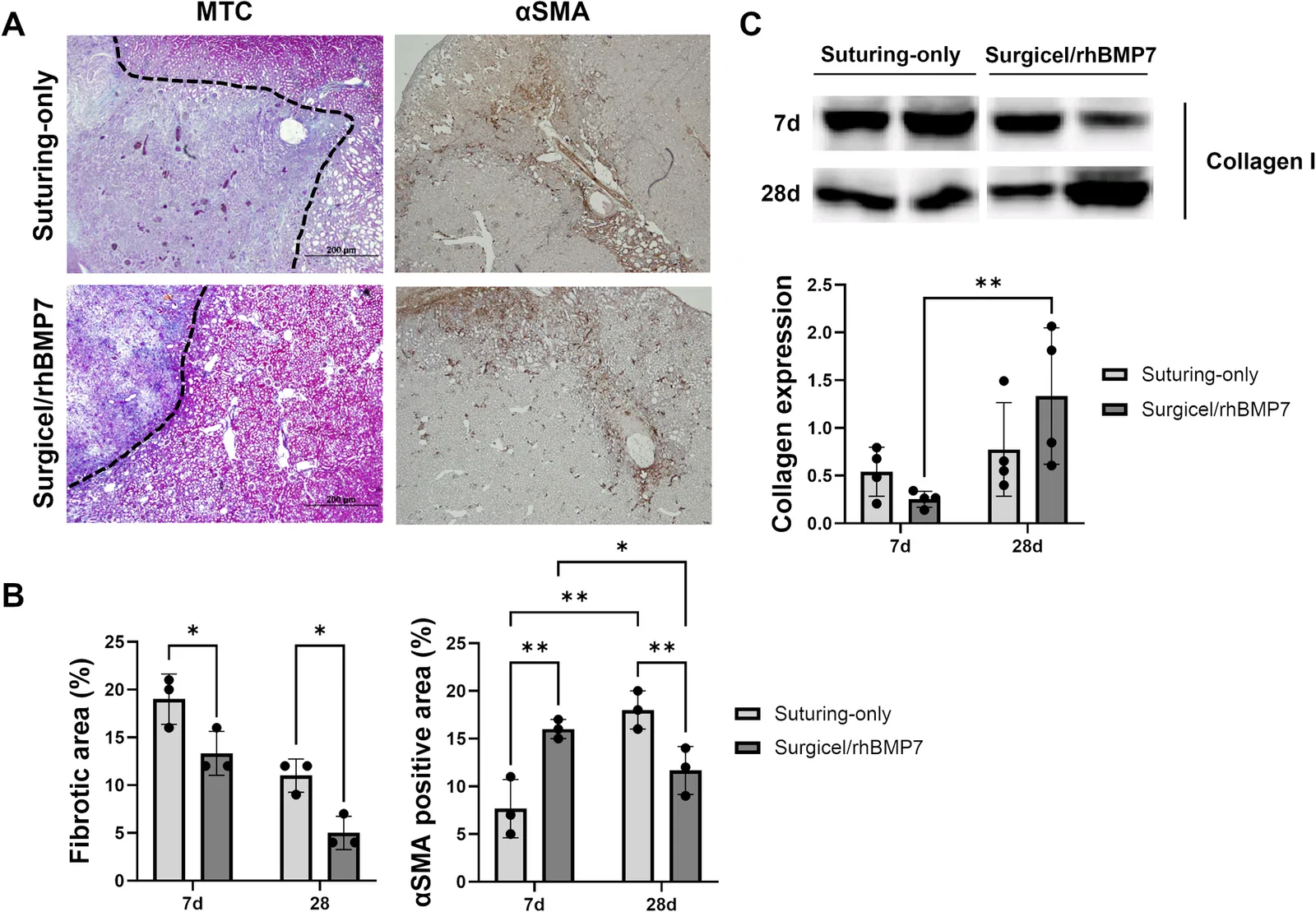
Fibrotic remodeling after surgically induced acute kidney injury. **(A)** Representative kidney sections stained for *α*-smooth muscle actin (α-SMA) and Mallory trichrome in the suturing-only and Surgicel/rhBMP-7-treated groups at 7 and 28 days after injury. The hatched line indicates the border between preserved and injured renal parenchyma. Scale bar: 200 µm. **(B)** Quantitative analysis of fibrotic area based on Mallory trichrome staining and *α*-SMA-positive area at 7 and 28 days after injury (*n* = 3 animals per treatment group at each time point). **(C)** Representative Western blots and densitometric analysis of collagen I expression in injured renal tissue at 7 and 28 days after injury (*n* = 4 animals per treatment group at each time point). Representative Western blot images were assembled from independent experiments performed at the indicated time points. Cropped images were derived from the original blots, and boundaries between non-adjacent lanes or cropped sections are indicated by dividing lines. Full-length uncropped blots and corresponding Ponceau S staining are provided in the Supplementary Information. Statistical significance: *p < 0.05, **p < 0.01 compared with the suturing-only group at the corresponding time point or between time points within the same treatment group (two-way ANOVA with Šídák’s multiple comparisons test for *α*-SMA and Mallory trichrome analyses; uncorrected Fisher’s LSD test for collagen I).

Mallory trichrome staining demonstrated a larger collagen-positive area in the suturing-only group than in the Surgicel/rhBMP-7 group at day 7 (*p* = 0.0236), with the same between-group pattern at day 28 (*p* = 0.0178) (Figures 7A,B).

α-SMA immunoreactivity was higher in the Surgicel/rhBMP-7 group at day 7 (*p* = 0.0041) but lower at day 28 than in the suturing-only group (*p* = 0.0183).

Western blot analysis showed increased collagen I expression at day 28 compared with day 7 in the Surgicel/rhBMP-7–treated group (*p* = 0.0081) (Figure 7C). Notably, increased collagen I abundance was not paralleled by greater histologically detectable fibrosis or *α*-SMA expression.

## 4 Discussion

The outcome of acute kidney injury depends not only on the severity of the initial injury, but also on the quality of subsequent repair processes, which determine progression toward regeneration or fibrosis (1, 14, 15). In this study, local application of Surgicel/rhBMP-7 was associated with changes in proliferative activity, markers associated with repair, and fibrotic remodeling in an experimental model combining ischemia and mechanical injury. These observations are consistent with previous studies conducted in experimental models of AKI using systemic administration of BMP-7, while extending these findings to a surgically relevant model using localized delivery (10, 11).

BMP-7 is a well-established regulator of renal development and epithelial homeostasis (16–18). Previous studies have reported antifibrotic and pro-regenerative effects of exogenous BMP-7 in experimental models of kidney injury (11, 12, 19). In the present study, increased phosphorylation of Smad1/5/8 at later time points in the treated group is compatible with differences in BMP-associated signaling. These findings are compatible with a potential role of BMP-7 in modulating post-injury responses (20).

Tubular epithelial repair after AKI is thought to rely primarily on proliferation of surviving differentiated cells (21, 22). In the present study, increased numbers of PCNA-positive tubular cells were observed in the Surgicel/rhBMP-7–treated group at both early and later time points. Expression of Pax2, a marker associated with injury-induced dedifferentiation, did not differ between groups, suggesting that increased proliferative activity was not accompanied by measurable changes in this aspect of the injury response (23, 24).

In parallel, Wnt-4 immunoreactivity remained higher in the Surgicel/rhBMP-7 group at day 28, indicating persistence of the observed Wnt-4-associated response (25–27). Together with the increased proliferative activity and the absence of significant between-group differences in Pax2 immunoreactivity, these findings suggest a coordinated pattern of proliferative and repair-associated responses. However, persistent Wnt-4 expression has also been associated with maladaptive repair and progressive fibrosis in certain experimental settings, warranting cautious interpretation of the sustained signal observed at day 28. Previous studies have highlighted the temporal dependence of tubular regenerative programs, whereby transient activation may support epithelial recovery, whereas persistent activation may accompany maladaptive repair and fibroproliferative responses (28). In this context, the sustained Wnt-4 immunoreactivity observed in the present study, together with increased collagen I abundance at day 28, raises the possibility that later repair-associated responses may not be exclusively regenerative.

Fibrotic remodeling represents a key determinant of long-term outcome after AKI (15, 29). In the current model, a reduced fibrotic area was observed in the Surgicel/rhBMP-7–treated group at both analyzed time points. *α*-SMA expression showed a time-dependent pattern, with higher levels at the early time point and lower levels at the later time point compared with suturing-only group. In parallel, collagen I expression increased at the later time point in the treated group without a corresponding increase in histomorphometric fibrosis. Importantly, this increase in collagen I should not be interpreted as a favorable outcome of renal repair. The apparent discrepancy between collagen abundance and morphometric fibrosis suggests that these parameters may reflect different aspects of tissue remodeling rather than identical biological processes. The biological basis and potential functional significance of this observation remain unclear and warrant further investigation.

Previous experimental models of surgical renal injury, including partial nephrectomy and incision-based approaches, have been used to study repair responses in the kidney (30, 31). The experimental model used in this study combines ischemia–reperfusion injury with direct parenchymal injury, approximating conditions encountered during nephron-sparing surgery and renal trauma. The present findings demonstrate that localized application of the Surgicel/rhBMP-7 formulation was associated with measurable biological responses in this context. This approach may be relevant from a translational perspective, as it enables spatially restricted delivery while potentially limiting systemic exposure. Accordingly, the intended translational context is renal surgery rather than local treatment of nonsurgical AKI.

Several limitations should be considered. A sham-operated group was not included, which limits comparison of the observed responses with uninjured renal tissue. In addition, the present analysis does not include a Surgicel-only comparator; therefore, the specific contribution of rhBMP-7 cannot be fully separated from potential effects of the carrier. Accordingly, the findings should be interpreted as reflecting the biological response to the combined local Surgicel/rhBMP-7 treatment. The release kinetics and local retention of rhBMP-7 and the resorption profile of the Surgicel carrier were not characterized; therefore, the present study does not establish an optimized dosing regimen or define a controlled-release profile for this formulation.

Overall, the present findings indicate that combined local Surgicel/rhBMP-7 delivery was associated with measurable biological responses following surgically induced AKI. These findings support further investigation of this localized delivery strategy for modulating renal repair after surgical kidney injury.

## Data Availability

The original contributions presented in the study are included in the article/Supplementary Material, further inquiries can be directed to the corresponding author.
