# Supplementary material for: Local Surgicel/rhBMP-7 delivery is associated with renal repair responses after surgically induced acute kidney injury
Source: Front Med (Lausanne). 2026 Sep 9;13:1941191. doi: 10.3389/fmed.2026.1941191 (PMC13597421; doi:10.3389/fmed.2026.1941191)
Supplement: Supplementary file 1 [file Supplementaryfile1.pdf]

Table S1. List of primary and secondary antibodies used for the immunohistochemical (IHC) and western blot (WB) analyses.

**Antibodies**	**Dilution**	**Manufacturer (Reference number)**
Mouse monoclonal anti-α-SMA	1:1000 (IHC)	Santa Cruz Biotechnology (sc-47778)
Rabbit polyclonal anti-BMP7	1:200 (IHC)	Abcam, Cambridge, UK (ab93636)
Mouse monoclonal anti-Pax2	1:400 (IHC)	Abcam, Cambridge, UK (ab79389)
Mouse monoclonal anti-PCNA	1:200 (IHC)	Merck Millipore (MAB377)
Rabbit polyclonal anti-Wnt4	1:200 (IHC)	Abcam, Cambridge, UK (ab150596)
Mouse monoclonal Collagen (COL1A)	1:3000 (WB)	Santa Cruz Biotechnology (sc-59772)
Rabbit polyclonal anti-phospho Smad1/5/8	1:200 (IHC)	Abcam, Cambridge, UK (ab1791)
Biotinylated goat anti-mouse	1:200 (IHC)<br>1:3000 (WB)	Thermo Fisher Scientific (A16070)
Biotinylated goat anti-rabbit	1:200 (IHC)	Thermo Fisher Scientific (65-6140)

Abbreviations: α-SMA, α-Smooth Muscle Actin; BMP7, Bone Morphogenetic Protein 7; Pax2, Paired Box Gene 2; PCNA, Proliferating Cell Nuclear Antigen; Smad, Suppressor of Mothers Against Decapentaplegic; Wnt4, Wingless-Type MMTV Integration Site Family, Member 4.

Figure S1

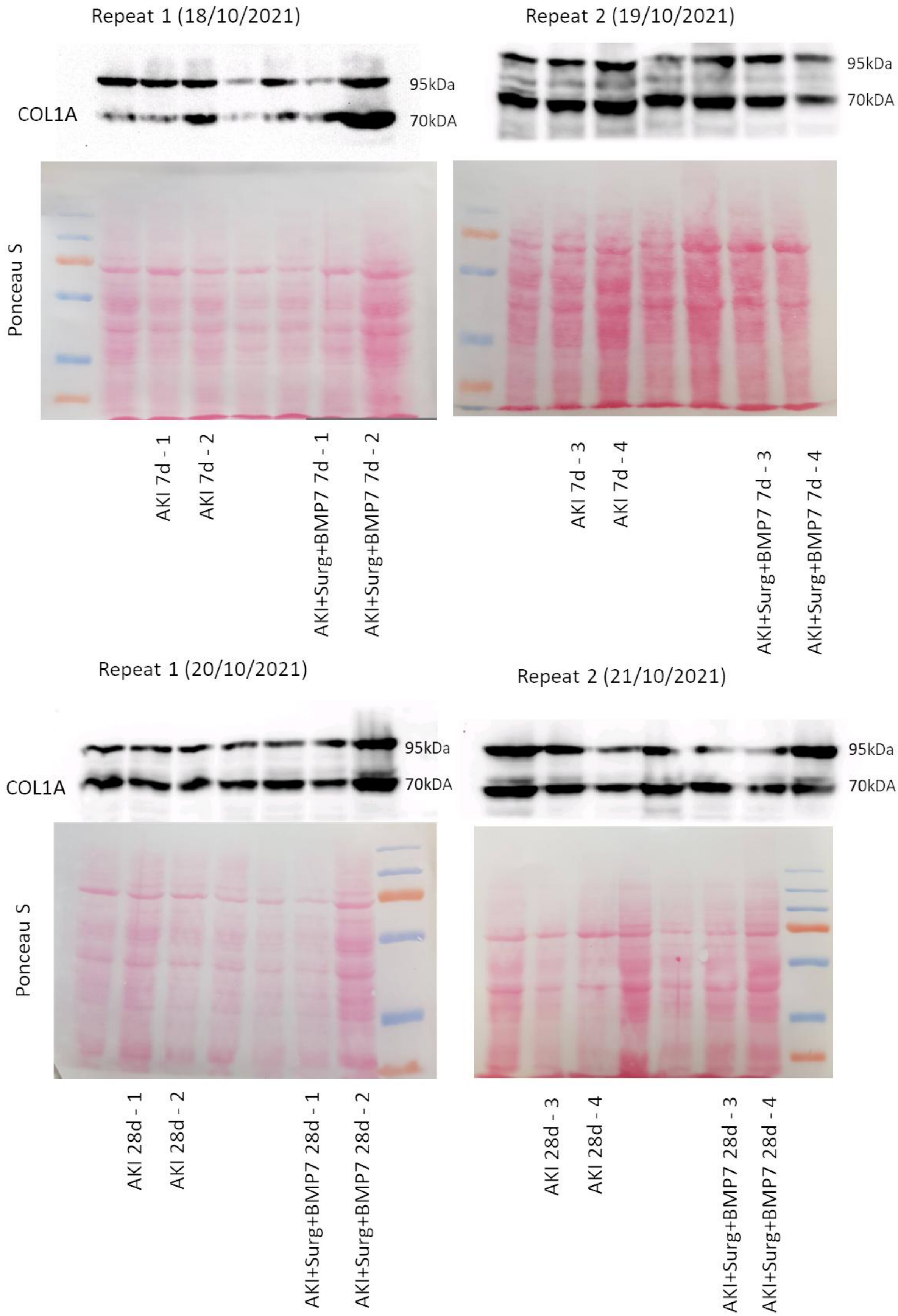

Repeat 1 (18/10/2021)
Repeat 2 (19/10/2021)
95kDa
70kDA
COL1A
Ponceau S
AKI 7d - 1
AKI 7d - 2
AKI+Surg+BMP7 7d - 1
AKI+Surg+BMP7 7d - 2
AKI 7d - 3
AKI 7d - 4
AKI+Surg+BMP7 7d - 3
AKI+Surg+BMP7 7d - 4
Repeat 1 (20/10/2021)
Repeat 2 (21/10/2021)
AKI 28d - 1
AKI 28d - 2
AKI+Surg+BMP7 28d - 1
AKI+Surg+BMP7 28d - 2
AKI 28d - 3
AKI 28d - 4
AKI+Surg+BMP7 28d - 3
AKI+Surg+BMP7 28d - 4
